# Effects of resource variation during early life and adult social environment on contest outcomes in burying beetles: a context-dependent silver spoon strategy?

**DOI:** 10.1098/rspb.2013.3102

**Published:** 2014-06-22

**Authors:** Paul E. Hopwood, Allen J. Moore, Nick J. Royle

**Affiliations:** 1 Centre for Ecology and Conservation, Biosciences, College of Life and Environmental Sciences, University of Exeter, Cornwall Campus, Penryn TR10 9EZ, UK; 2 Department of Genetics, University of Georgia, Athens, GA 30602, USA

**Keywords:** silver spoon, environmental-matching, social environment, resource holding potential, developmental effects

## Abstract

Good early nutritional conditions may confer a lasting fitness advantage over individuals suffering poor early conditions (a ‘silver spoon’ effect). Alternatively, if early conditions predict the likely adult environment, adaptive plastic responses might maximize individual performance when developmental and adult conditions match (environmental-matching effect). Here, we test for silver spoon and environmental-matching effects by manipulating the early nutritional environment of *Nicrophorus vespilloides* burying beetles. We manipulated nutrition during two specific early developmental windows: the larval environment and the post-eclosion environment. We then tested contest success in relation to variation in adult social environmental quality experienced (defined according to whether contest opponents were smaller (good environment) or larger (poor environment) than the focal individual). Variation in the larval environment influenced adult body size but not contest success *per se* for a given adult social environment experienced (an ‘indirect’ silver spoon effect). Variation in post-eclosion environment affected contest success dependent on the quality of the adult environment experienced (a context-dependent ‘direct’ silver spoon effect). By contrast, there was no evidence for environmental-matching. The results demonstrate the importance of social environmental context in determining how variation in nutrition in early life affects success as an adult.

## 1. Introduction

Variation in nutrition experienced by individuals during development can have long-term effects on adult phenotype, such as body mass [1], fecundity [2], dominance status [3] and longevity [4] that directly affect the fitness of individuals. Furthermore, early-life developmental effects on phenotypes may also lead to indirect effects that can impact on the expression of traits in other individuals (e.g. via indirect genetic effects [5] or epigenetic effects [6]) and influence the population dynamics and evolutionary trajectories of organisms [7,8].

Nutritional variation during early development is hypothesized to affect fitness in different ways depending on the quality of the subsequent adult environment experienced [9,10]. For example, an advantage in adulthood for individuals with plentiful early developmental resources over those that experienced poorer early conditions, regardless of their adult environment, is known as a ‘silver spoon’ effect [9,10]. Alternatively, the ‘environmental-matching’ hypothesis predicts individuals whose adult environment ‘matches’ that which they experienced during development will have the highest fitness [10]. In environmental-matching, phenotypic attributes expressed as a result of poor developmental conditions are purported to ‘program’ an individual to deal with correspondingly poor conditions in adulthood such that in these circumstances they even outperform individuals that experienced better developmental conditions [10,11]. While there is empirical support for silver spoon effects in general [2,12–15] and even environmental ‘mismatching’ [16] there is little or no clear empirical support for the environmental-matching hypothesis (but see [17,18]).

Lack of support for environmental-matching may reflect the fact that in nature the quality of the environment that individuals experience is likely to be primarily determined by their competitive ability in relation to the competitive ability of other individuals in the population (i.e. the social environment), rather than resource abundance *per se* (i.e. the physical environment). Variation in the abundance of food, for example, may not affect all members of the population equally if individuals also vary in their competitive ability, which determines their access to such resources. In addition, the particular social environment experienced by individuals (e.g. the sex and/or competitive ability of conspecifics) can also impact the expression of traits [5], and developmental conditions themselves may evolve through changes in the population social environment [8]. However, overall levels of competition for resources will be affected by the abundance of those resources, so there is considerable feedback between the physical environment and the social environment, which influences the quality of environments, that individuals experience. Despite the importance of social context in determining the quality of adult environments, a recent review by Monaghan [10] showed that the majority of studies that test the effects of variation in resource availability early in life on subsequent adult traits define adult environment quality in terms of food abundance and do not consider the social environment.

In addition to not measuring environmental quality in an appropriate context another reason why environmental-matching may be poorly supported is because the early developmental environment is often loosely defined, including any or all of the period between conception and developmental maturity; an approach that implicitly assumes that effects of variation in nutrition on adult phenotype will be largely independent of when they occur during development. Recent studies have demonstrated the importance of the timing of nutritional deprivation on success in adulthood [19,20], which may have independent or interactive effects on the expression of adult phenotypes. Distinct stages may often exist in the developmental processes of organisms when nutritional variation has disproportionately large effects on phenotypic expression [21], including effects on disease susceptibility in later life [22] and adaptive polyphenisms, such as the winged phase switch in pea aphids, *Acyrthosiphon pisum* [23] or horn elongation in dung beetles, e.g. *Onthophagus taurus* and *O. acuminatus* [24,25]. These ‘developmental windows’, although potentially important, can be difficult to identify because developmental progression may involve multiple critical windows with interacting effects on the expression of phenotypes [26,27]. The impact of variation in nutrition early in life versus later in life may also be hard to quantify because many species experience fluctuations in availability of nutrition throughout post-natal, juvenile and adult development. For example, in many seabirds pre-reproductive development may extend over several years including pre-natal, post-hatching, pre-independent and juvenile (pre-reproductive) stages [28] and early experience may be correlated with later experience due to carry-over and cohort effects [15,29].

We previously identified a key developmental window in the burying beetle *Nicrophorus vespilloides* during which variation in food availability determined later success as an adult in contests for breeding resources [19]. This developmental window occurs during the period when beetles are undergoing reproductive maturation, and may last less than a week from the time the mouthparts of an eclosed beetle have sclerotized and feeding commences (approx. 36 h post-eclosion) until viable matings can take place [19]. Burying beetles feed on putrescent carrion and various invertebrates so variation in food availability in the wild is likely to occur because of rainfall and temperature fluctuations affecting beetle (and potential prey) activity, and through stochastic availability of carrion. However, this post-eclosion nutritional bottleneck is not the only window during which variation in nutrition may have long-term effects on adult traits; the social and nutritional environment experienced during larval development is also important [30].

In burying beetles, the impact of the adult social environment on individual fitness is particularly important because a fundamental aspect of their life cycle involves direct contests for breeding resources (vertebrate carcasses). Success in contests over suitable breeding resources in *N. vespilloides* is closely related to reproductive success in both sexes [31]. A single small carcass (e.g. mice, shrews, small songbirds) is the sole resource necessary to rear a brood of offspring. Males that locate a carcass but are unable to become the dominant male may adopt a subordinate strategy and sneak copulations with females; likewise, any female unable to secure the dominant female position may parasitize a brood by laying eggs nearby [31]. However, parentage proportion in broods is lower for subordinates compared with the dominant pair [32]. Thus, from an individual's perspective, its own relative size among competitors at a carcass characterizes the quality of the reproductive environment [33,34]. Moreover, it is possible that the body size of an individual (related to the quality of its developmental environment), might itself act as an intrinsic cue of social environmental quality, i.e. whether it is likely to be larger or smaller than competitors. For example, experimental evidence in a congener, *N. orbicollis,* suggests that small males may preferentially employ an alternative reproductive tactic spending proportionally more time trying to call females with pheromones (with whom they mate in the absence of a carcass) than do large males [35].

**Figure 1.**
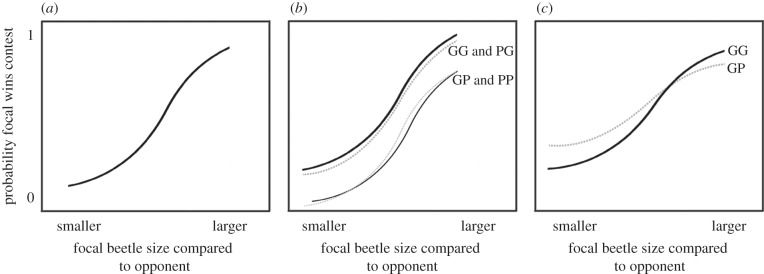
Graphical representation of key predictions. *Y*-axis: mean probability of victory for focal beetles, *x-*axis: ‘smaller’ to ‘larger’ = increasing relative size advantage of focal beetle over opponent. (*a*) Contest success probability depends on the relative size of opponent; (*b*) silver spoon effect of post-eclosion treatment but not larval treatment (i.e. GG and PG treatment groups have a higher probability of success than GP and PP groups for any given adult environment experienced (relative size compared with opponent)); (*c*) environment-matching for post-eclosion delayed-feeding early-life environment (i.e. individuals in the GP treatment group fare relatively better than GG beetles in when the adult environment is poor).

In this study, we test for silver spoon and environmental-matching effects by manipulating the early nutritional environment of *N. vespilloides*. In contrast to most previous studies that treat early development as a single, largely homogeneous juvenile stage, we independently manipulated larval and post-eclosion pre-reproductive nutritional windows. This is because good nutrition during the first (larval) window, if considered alone, might produce relatively large adults endowed with a putative silver spoon competitive advantage in proportion to their relative size, independent of later environmental conditions. Similarly, if relative size was controlled for and variation in post-eclosion nutrition considered alone, any advantage might also suggest a silver spoon effect. However, potential interactions between these developmental windows would be missing, e.g. smaller individuals might bear food shortage better than larger individuals. Furthermore, contests only take place in the presence of a large enough carcass on which to breed, so variation in the quality of the environment is primarily determined by variation in the social environment in which any contests take place (i.e. relative competitive ability of individuals). Variation in the relative size of competing individuals therefore represents variation in the quality of the adult environment experienced by focal individuals. We produced four different early environment treatments: (GG = control: standardized ‘good’ laboratory conditions throughout development (reared on a large carcass as larvae and ad libitum food post-eclosion); GP = good larval environment (large carcass) + poor (delayed-feeding) post-eclosion environment; PG = poor larval environment (small carcass) + good post-eclosion environment (i.e. ad libitum feeding immediately from eclosion); PP = poor larval environment + poor post-eclosion environment). This allowed us to quantify the effects of different combinations of dietary manipulation treatments on adult competitive ability in *N. vespilloides* (i.e. success in competition for carcasses) for both males and females. It is known that variation in larval nutrition has permanent effects on an individual's body size [30], and that larger individuals are more successful in contests for carcasses than smaller beetles [30,34], so we predicted a positive relationship between the relative size of a focal individual compared to its opponent and the probability of contest success (figure 1*a*). Thus, the adult (social) environment was defined as good when focal beetles were larger than their opponent, or poor when focal beetles were smaller than their opponent. Competitors for carcasses vary in number and size in the wild [32], so the probability of encountering a ‘good’ contest environment is lower for relatively small beetles.

A clear silver spoon effect would be supported if individuals that have experienced good larval and/or good post-eclosion treatments always do better than individuals that experienced poor larval and/or poor post-eclosion treatments for any given environment experienced in adulthood (i.e. will have higher elevation relationship between the probability of winning and body size difference). For example, if there is a silver spoon effect of post-eclosion delayed-feeding we would expect to see animals in the GG and PG treatment groups having greater probability of success in contests than those in the GP and PP groups, and if there is a silver spoon effect of a good larval treatment GG and GP will have higher probability of success across social environments compared with beetles in the PG and PP treatments (e.g. figure 1*b*).

By contrast, environmental-matching would be supported if there is a significant interaction between one or both of the nutritional treatments and the adult environment experienced, i.e. individuals that experienced poor early-life environments should have greater probability of success than beetles that had good early-life environments when the adult environment they experience is poor (i.e. when they are smaller than their opponent; figure 1*c*).

## 2. Material and methods

### (a) Larval nutritional environment

Over 300 wild beetles were caught in funnel-type bottle traps baited with putrescent salmon in Devichoys wood in Cornwall, UK (SW 772 376) during the summer of 2012. Beetles were maintained and bred for four generations in accordance with the methods of Head *et al*. [36]. Three weeks after beetles eclosed, 66 virgin adult males and 66 virgin adult females were drawn from this outbred F_4_ stock population and randomly allocated to one another to form breeding pairs. The experimental design involved a 2 × 2 factorial manipulation of larval and post-eclosion nutritional environments as follows: for manipulation of the larval environment, 33 of the 66 pairs were allocated a ‘standard’-sized mouse carcass of 20.76 ± 0.05 g (i.e. good larval environment, ‘G_’) and 33 pairs a smaller carcass weighing 5.31 ± 0.05 g (i.e. poor larval environment, ‘P_’) for use as their single available breeding resource. In total, 1511 offspring were raised from the combined 66 pairs in both larval environments. These larval nutritional environments were chosen because these carcass sizes fall into the range expected in the wild (juvenile and adult small mammals and birds) and are within the range of carcass sizes that are commonly used for breeding by *N. vespilloides* in the laboratory [37–39].

### (b) Post-eclosion nutritional environment

**Figure 2.**
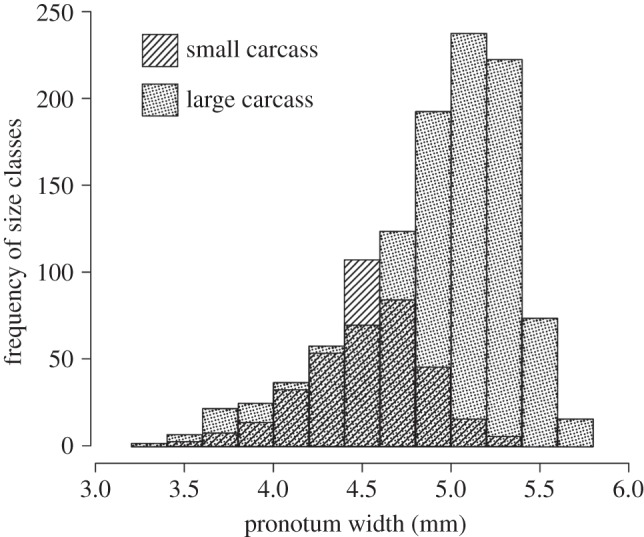
Histogram showing size distribution of all adult experimental individuals (*n* = 1511) from 33 families reproducing on a small carcass (poor larval environment; diagonal lines) and 33 families reproducing on a large carcass (good larval environment; dots).

As previously reported by Bartlett & Ashworth [30], we found that broods reared on small carcasses (i.e. poor larval environment) consisted of individuals with smaller average size than did broods reared on larger carcasses (figure 2). The underlying size difference between adults reared under different larval treatments (carcass size) was controlled to ensure that focal beetles experiencing poor larval environments (i.e. PP or PG treatment groups) did not have a higher probability of meeting an opponent larger than themselves than beetles in the good larval environment treatments groups (GP or GG). This was achieved by daily assigning all beetles eclosing from the poor larval environment to size classes (pronotum width to the nearest 0.2 mm) then matching the number in each size class with individuals from good larval environments. Excess individuals, i.e. those from either larval environment with insufficient numbers of a particular size class from the alternative larval environment, were discarded (*n* = 791). Sufficient stock beetles were also drawn to match daily size class numbers for use as competitive trial opponents. The remaining 719 newly eclosed beetles were allocated at random between one of two post-eclosion diets. In the first post-eclosion diet group, newly eclosed individuals were not fed for the first 6 days post-eclosion (*n* = 373); this delayed-feeding diet (‘_P’) occurred during their maturation developmental window [19]. After their fast, this group was fed using our standard ad libitum feeding regime of two decapitated mealworm larvae, *Tenebrio molitor*, twice weekly). The second post-eclosion diet group (*n* = 346), by contrast, were fed mealworms immediately following eclosion and ad libitum thereafter (‘_G’). Beetles from this second group that were both reared on larger mice and fed ad libitum at eclosion (‘GG’) effectively constituted controls that received good nutrition throughout development. By contrast, ‘PP’ individuals experienced poor nutritional environments through development. ‘PG’ individuals had poor larval but good post-eclosion environments, whereas ‘GP’ individuals experienced good larval but poor post-eclosion environments.

### (c) Competitive trials

A random sample of 25 animals (of both sexes) was taken from each of the four treatment groups to be used in competitive trials (i.e. total of 100 focal beetles). Stock beetles that had received standard rearing conditions throughout development (i.e. they were equivalent to the ‘GG’ treatment) were used as contest opponents and chosen haphazardly to eliminate bias in the direction of size differences between opposing beetles. One hundred independent intra-sexual contests were staged and filmed in the laboratory in a naturalistic set-up in Nicrocosms (see the supplementary information for details in ref. [19]). These arenas facilitate observation of recorded video footage of individual beetle interactions over the whole pre-natal period, during which time conflicts are resolved and dominance status of individuals is established. In each Nicrocosm, single focal beetles, males or females, were placed simultaneously on a fresh carcass with a same-sex opponent and a stock breeding partner (i.e. a male in female–female contests and a stock female in male–male contests), during the afternoon beetle activity period between 14.00 and 17.00. The dominant individual in male–male and female–female contests was defined as the beetle that secured the carcass and succeeded in partnering with a stock individual of the opposite sex to process the carcass [19].

### (d) Statistics

All analyses were performed using ‘R’ v. 2.14.1 [40]. The effect of larval environment (carcass size—large or small) on mean (within brood) adult body size was analysed using a linear model with carcass size, maternal size and paternal size as explanatory variables. Mean larval number produced per brood had a bimodal distribution and was analysed using the Wilcoxon rank sum test grouped by carcass size (large or small). The effects of the experimental larval and post-eclosion nutritional environments experienced by individuals (2 × 2 factorial) on the probability of success in competitive trials (contest success) were analysed using a general linear model using a quasi-likelihood approach (quasi-binomial) to account for overdispersion [41]. We included the adult (social) environment experienced (relative size of focal individual compared to its opponent) and sex as variables, testing for all two-way interactions. The relative difference between the size of the focal beetle and its competitor was quantified using the following equation: 1 – (opponent size/focal size). This controlled for differences in the absolute size of pairs of beetles across treatments. However, our results are not dependent on this particular measure of relative size. The same terms were significant when analyses were run using absolute size difference, i.e. by subtracting the pronotal width of the focal beetle from that of its opponent. Unless stated otherwise, means are presented ±1 s.e. throughout.

## 3. Results

### (a) Effects of larval nutritional treatment on brood number and body size

Adult body size was strongly positively related to the larval environment experienced (i.e. carcass size). Parents produced small larvae on small carcasses: brood mean offspring size (pronotum width as adult) from large carcasses = 4.95 ± 0.03 mm; offspring size on small carcasses = 4.55 ± 0.04 mm (LM, *F*_1,62_ = 64.025, *p* < 0.0001; figure 2). Controlling for the effects of carcass size there was no statistically significant effect of maternal size (*F*_1,61_ = 1.382, *p* = 0.244), paternal size (*F*_1,61_ = 2.542, *p* = 0.116) or the interaction between them (maternal size × paternal size, *F*_1,59_ = 0.037, *p* = 0.848) on the size of offspring in adulthood.

Fewer larvae per brood were successfully reared on small carcasses (small carcasses: 12.77 ± 0.60; large carcasses: 33.85 ± 1.43; Wilcoxon rank sum test, *W* = 993.5, *n* = 64, *p* < 0.0001) and overall, fewer than 10% of individuals reared on small carcasses attained even the average size (i.e. 4.95 mm pronotum) of those reared on large carcasses.

### (b) Effects of early-life nutritional treatment and adult social environment on success in contests

**Figure 3.**
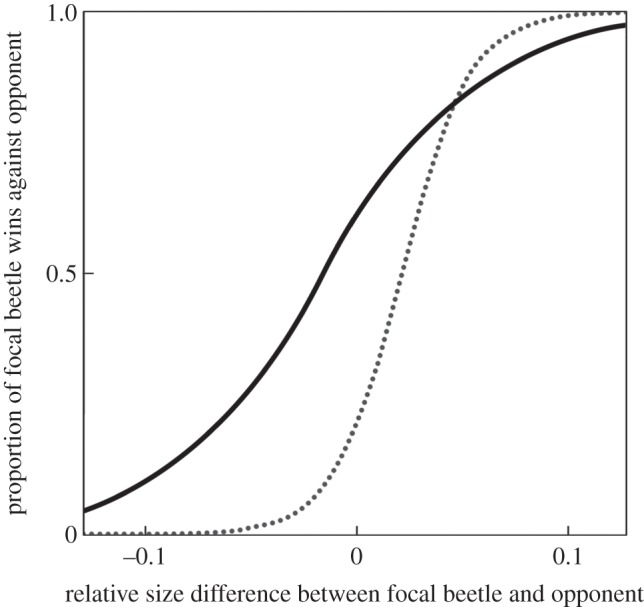
Model fit of relationship between adult environment experienced by focal individuals (relative size difference) on the *x-*axis and probability of winning a contest on the *y-*axis. Solid line = beetles with ‘good’ post-eclosion nutritional environment (i.e. fed ad libitum post-eclosion (GG and PG)); dashed line = beetles with ‘poor’ post-eclosion nutritional environment (i.e. post-eclosion delayed-feeding (GP and PP)).

There was a significant interaction between the quality of the adult social environment (relative size of focal beetle compared with opponent) and the post-eclosion nutritional environment experienced that determined the probability of success in contests (GLM, quasi-binomial errors, relative size difference × post-eclosion environment, *F*_1,96_ = 4.244, *p* = 0.042; figure 3). Beetles that experienced a poor post-eclosion environment were more sensitive to the adult environment they experienced than individuals that had a good post-eclosion nutritional environment (the relationship had a steeper slope, figure 3). They also had a lower probability of success in contests across adult environments, unless they were considerably larger than their opponent (i.e. lower elevation of the relationship, figure 3). All other two-way interactions were non-significant (all *p* > 0.12) and neither sex (sex, *F*_1,95_ = 1.371, *p* = 0.245) nor larval environment *per se* (i.e. independent of its effect on adult size) were significant predictors of contest success (larval environment, *F*_1,95_ = 0.003, *p* = 0.956). However, there was a significant main effect of the adult social environment experienced by individuals on contest outcome (relative size difference between focal beetle and its opponent: *F*_1,97_ = 54.867, *p* < 0.0001), with focal beetles being more successful the larger they were relative to their opponent as expected. There was also a non-significant trend for beetles experiencing ‘poor’ post-eclosion (delayed-nutrition) environments to be less likely to win contests than those reared in ‘good’ post-eclosion environments (post-eclosion nutritional environment, *F*_1,97_ = 3.260, *p* = 0.074).

## 4. Discussion

We manipulated the nutritional environment experienced by burying beetles *N. vespilloides* during two different developmental windows; first during larval development then, after pupation, during the post-eclosion maturation stage. We tested whether variation in early-life environments, at either or both of these developmental stages, predicted the probability of success during contests for breeding resources when the adult social environment also varied in quality between poor (focal individuals were small compared to their opponent) and good (focal individuals were larger than their opponent). We found a significant interaction between the post-eclosion nutritional environment experienced during development and adult social environment (size relative to opponent) predicted the probability of success in contests: the steeper slope relationship (figure 3) of beetles that experienced poor post-eclosion nutrition indicates that they had a lower probability of winning contests across adult social environments than did beetles that experienced good post-eclosion nutrition, except when they were substantially larger than their opponents (see also [19]). However, the effect of the larval environment (carcass size) was also important in determining the probability of winning contests for breeding resources because the size of carcass that individuals were reared on determined size at adulthood, and therefore the probability of encountering a larger opponent. As a result, although the larval environment does not affect the probability of winning a contest for a given social environment experienced in adulthood, it does affect the probability of experiencing a poor-quality adult environment (i.e. encountering an opponent larger than itself), which in turn affects contest outcome. There were therefore both direct (post-eclosion environment) and indirect (larval environment) effects of variation in nutrition during development on the probability of winning contests in adulthood.

In burying beetles reproduction depends upon finding and securing access to a carcass of a small vertebrate. As the availability of carcasses suitable for breeding is likely to be limited and their distribution ephemeral, direct contests over breeding resources are common [31]. Body size and condition of the individual are known to be important determinants of success in contests [19,30,34], so the quality of the adult (social) environment that individuals experience can be defined in terms of their size compared with that of their opponents, from good (larger than opponent) to poor (smaller than opponent). Studies of developmental nutritional variation at different life stages often look for interactions between juvenile and adult experience, but rarely, if ever, consider early-life effects on adult phenotypes expressed in the context of social competition [10]. By independently manipulating the quality of the nutritional environment at two different stages of development and varying the quality of the adult social environment experienced, we could test whether early-life environments predict success in later competitive social environments. The question then is: do the data support an environmental-matching hypothesis or fit a silver spoon scenario?

### (a) Environmental-matching or silver spoon?

For environmental-matching to be supported, we would expect individuals that had a poor nutritional environment during the larval stage (individuals reared on small carcasses) and/or post-eclosion stage to be better relative competitors in poor adult environments (i.e. when smaller than their opponent, figure 1*c*). In addition, evidence for an adaptive strategy should fulfil the requirement that early environmental conditions during development reliably predict later environmental conditions. Pupating at a relatively small size might provide such a cue: there will be a higher likelihood of encountering a rival of greater size than itself. This would represent a potential serious disadvantage in securing or defending a breeding resource. However, we found no evidence that beetles that experienced poor early-life environments (i.e. beetles from the PG, GP or PP groups) had a greater relative probability of contest success when the adult environment was poor compared to beetles that had good nutritional environments throughout development (GG group; see also [42]). In our experiment, the probability of contest success for adults depended not only on the quality of the adult social environment (relative size of focal compared to opponent) but also on an interaction with the post-eclosion environment. However, the direction of the effect of the interaction was opposite to that predicted by environmental-matching. Individuals reared under poorer post-eclosion environments (_P) did even worse than those with good post-eclosion environments (_G) when adult environments were poor (i.e. from an adaptive perspective, these individuals would be environmentally mismatched, figure 3). There was therefore no evidence in support of environmental matching.

Did poor early nutrition disrupt optimal development leading to a silver spoon effect? Beetles experiencing good post-eclosion environments (access to ad libitum food: GG and PG treatment groups) had better relative performance during contests for a given quality of adult environment than did those that experienced post-eclosion delayed-feeding (PP and GP treatment groups; figures 1*b* and 3), except when adult environments were very good (i.e. when much larger than their opponent). This indicates a context-dependent, direct silver spoon effect.

In contrast to the effects of variation in nutrition experienced post-eclosion, variation in the nutritional environment experienced during the larval stage did not affect the probability of success during contests for a given quality of adult environment (i.e. controlling for body size differences). However, because body size is closely related to the quality of the larval nutritional environment there is likely to be a close association between competitive ability in adulthood and the size of the carcass on which individuals develop. Large size relative to opponents was a primary determinant of success in adult contests for breeding resources, as has also been documented in other studies of this species [19,30,34], and widely reported across other taxa [43–46].

Larvae developing on small carcasses were themselves small as adults (figure 2) and in nature small beetles are likely to occupy different parameter space with respect to the likely adult social environments they experience. In the wild, the importance of being small in contests for breeding resources may depend on the size distribution of individuals in the population. Assuming a normal distribution of the availability of carcass sizes in the wild, because broods reared on small mice contain smaller individuals and fewer individuals, the size distribution of adult beetles is likely to be skewed towards relatively large beetles. Consequently, larvae developing on small carcasses suffer a disproportionally high probability of encountering rivals of greater size than themselves. As a result, in addition to the direct silver spoon effects of variation in quality of the post-eclosion environment on contest success there are also indirect silver spoon effects on contest outcomes of variation in the nutritional environment experienced during the larval stage: larvae reared on small carcasses are not competitively inferior *per se* (i.e. for a given adult social environment), but are more likely to experience poor adult social environments (encounter an opponent larger than themselves) because they are small. However, both of these effects are context-dependent, with the direct effect only occurring when the adult environment is poor and the indirect effect only occurring when the adult environment is good, so neither can be considered simple, clear-cut silver spoon effects [2,12–15].

The advantage of large size may only be realized in the context of competition at a carcass. In *N. vespilloides*, larger males mating with polyandrous females have no advantage over smaller males when mating away from a carcass (i.e. sperm competition without any immediate male–male competition) but smaller males suffer a disadvantage when both males mated on a carcass [47]. When the population is dense and there are many competitors for carcasses larger individuals may thus have an advantage. Moreover, when suitable carcasses are more abundant, large individuals may be at an advantage by having the potential to produce more broods with greater numbers than can smaller beetles [48]. However, small beetles could mitigate their disadvantage in contest ability by avoiding contests, for example, by being the first beetle to locate a carcass and/or preferentially attracting females. If they are subsequently usurped from ownership of a carcass by a larger individual they may still resort to alternative reproductive strategies such as brood parasitism or satellite behaviour and/or return to the mating-pool early. There may also be direct general benefits of a smaller body size such as lower costs of flight and lower overall maintenance costs [49] that might enable wider or more prolonged searches for resources. Smaller individuals may not be at a reproductive disadvantage when carcasses are small or poor quality and intriguingly, there is evidence that they can produce broods with offspring substantially larger than themselves [30].

Fitness implications of offspring size and nutritional variation in burying beetles are unknown. In burying beetles, body size is mediated by the caring behaviour of parents as broods are tailored to match the size of the breeding carcass, i.e. fewer larger larvae, or more but smaller larvae may be reared [50,51]. This leads to the possibility that producing a brood of smaller (or larger) than average offspring might be a parental adaptive response to the likelihood of future hard times for their offspring. Van De Pol *et al*. [15] used a long-term dataset to study transgenerational effects of natal origin (high-quality or low-quality habitat patches) on fitness in wild oystercatchers, *Haematopus ostralegus*. By measuring fitness both as individual components (e.g. survival to adulthood and recruitment) and also combined components (e.g. relative output per fledgling from each habitat through subsequent breeding years) it emerged that long-term effects of the early environmental conditions were as important as short-term effects in this species. One of the drivers of the long-term effects was the increased likelihood that offspring reared on high-quality patches would themselves secure good breeding habitat as adults. However, burying beetles do not face the same choice between low- and high-quality breeding patches; all individuals must vary their tactics depending on whether a carcass is poor or good, and contested or uncontested. Nevertheless, their reproductive success depends on interactions between the effect of their early environment and the social environment they experience in adulthood. Unpredictability in both the nutritional and social environment, they face may help to explain the extraordinary variation in size and mating tactics in *N. vespilloides*.

## 5. Conclusion

Our results indicate that effects of variation in developmental conditions on success in adulthood may be complex and dependent on the ecological context in which they are expressed. There was no evidence for environmental-matching or simple silver spoon effects. Instead results showed that benefits of good nutrition during development depended on the adult social environment individuals experienced. Adult beetle size is permanently influenced by the size of the carcass on which individuals develop; beetles reared on small carcasses are smaller than beetles reared on larger carcasses. Body size is the most important predictor of contest success for breeding resources, so a large carcass represents an ‘indirect’ silver spoon for the brood that it supports. However, benefits of a good larval environment are dependent on the social environment that adults experience because beetles reared on smaller carcasses fared no worse against opponents than did those reared on larger carcases, for a given size differential between individuals. When beetles had good nutrition post-eclosion compared with a delay in feeding they benefitted from a ‘direct’ silver spoon effect on contest success. However their advantage was only evident when the adult environment was poor (i.e. they met an opponent larger than themselves). Being small is also not necessarily disadvantageous for burying beetles. Size-dependent success may be influenced by breeding resource unpredictability, population density, variation in the proportion of contested carcasses, sexual selection and carcass discovery, all of which need to be investigated in the wild. These factors may be key to understanding the evolution and maintenance of the plasticity in body size and reproductive strategy found in *N. vespilloides*.

## Data accessibility

The data have been deposited in the Open Research Exeter (ORE) data repository, and are freely available at http://hdl.handle.net/10871/14720.
